# Supplementary material for: In Vitro Probiotic Characteristics and Whole Genome Sequence Analysis of Lactobacillus Strains Isolated from Cattle-Yak Milk
Source: Biology (Basel). 2021 Dec 29;11(1):44. doi: 10.3390/biology11010044 (PMC8772927; doi:10.3390/biology11010044)
Supplement: Supplementary file 1 [file biology-11-00044-s001.zip › biology-1484092-supplementary.pdf]

Figure.S1 Gram staining of lactic acid bacteria

Figure.S2 Hemolytic activity of LAB

Figure.S3 Phylogenetic tree of C12

Figure.S1

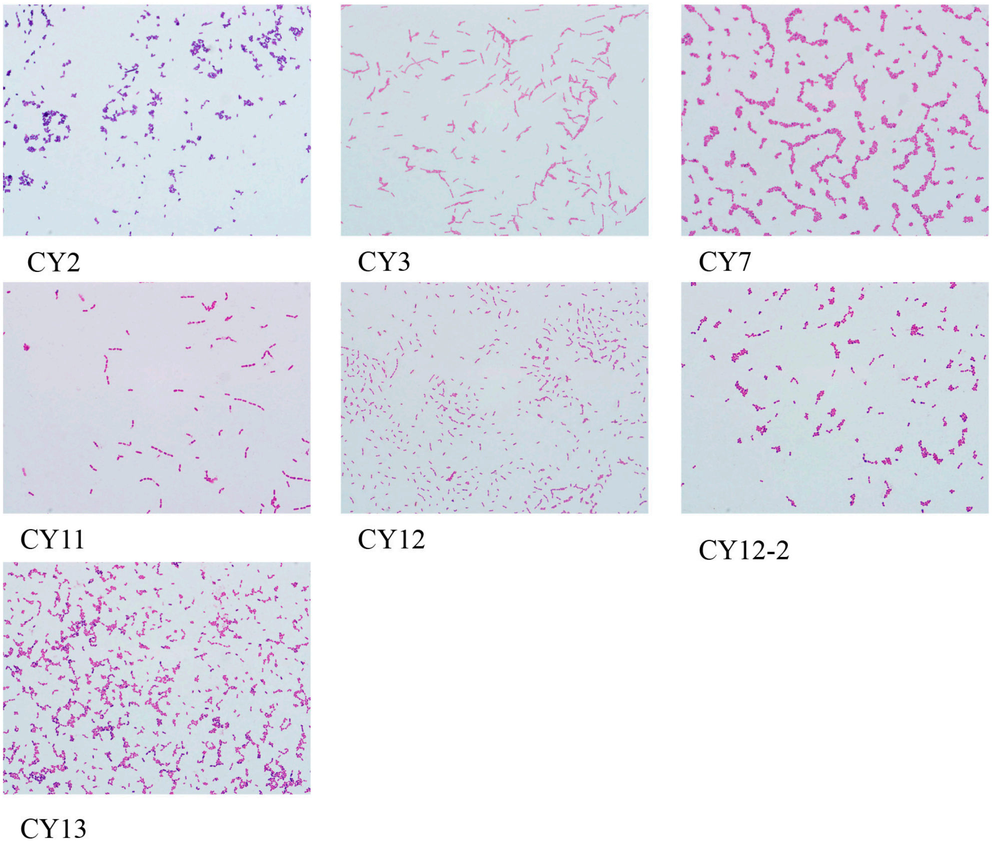

Figure.S2

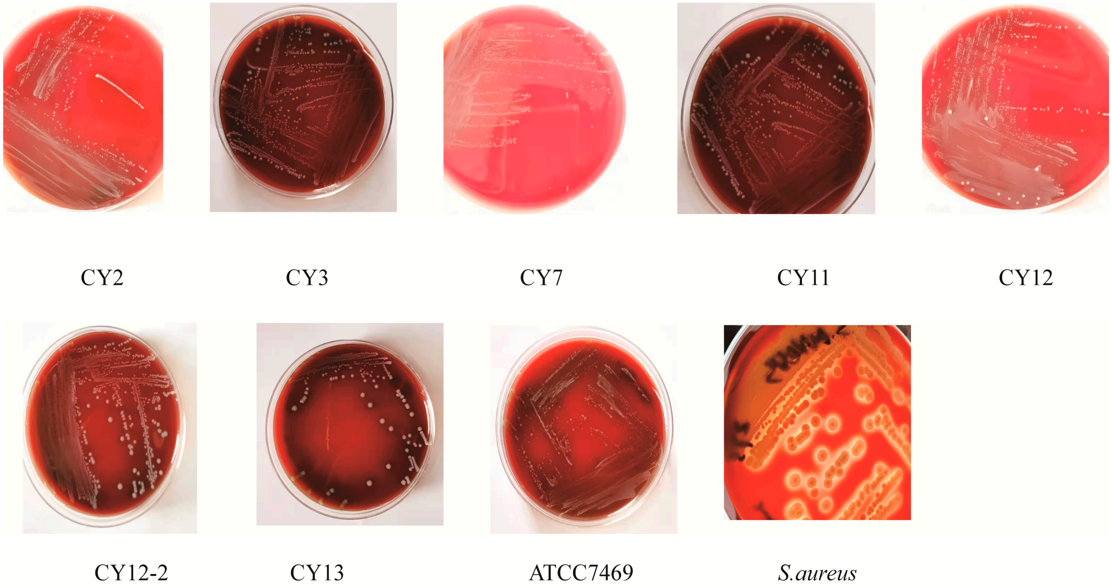

Figure.S3

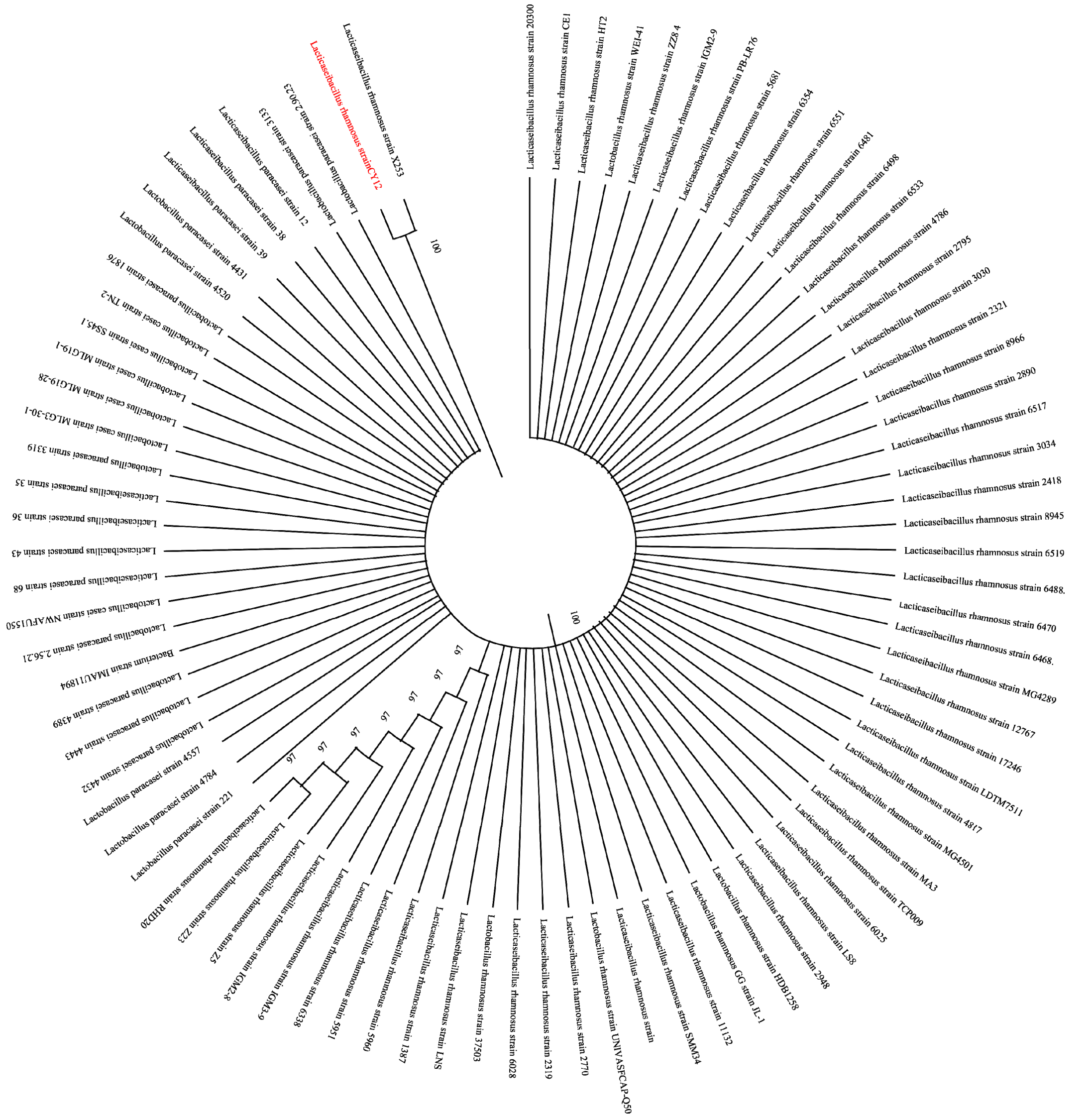
Lacticaseibacillus rhamnosus strain 20300
Lacticaseibacillus rhamnosus strain CE1
Lacticaseibacillus rhamnosus strain HT2
Lactobacillus rhamnosus strain WEI-41
Lacticaseibacillus rhamnosus strain ZZ8.4
Lacticaseibacillus rhamnosus strain IGM2-9
Lactobacillus rhamnosus strain PB-LR76
Lacticaseibacillus rhamnosus strain 5681
Lacticaseibacillus rhamnosus strain 6354
Lacticaseibacillus rhamnosus strain 6551
Lacticaseibacillus rhamnosus strain 6481
Lacticaseibacillus rhamnosus strain 6498
Lacticaseibacillus rhamnosus strain 6533
Lacticaseibacillus rhamnosus strain 4786
Lacticaseibacillus rhamnosus strain 2795
Lacticaseibacillus rhamnosus strain 3030
Lacticaseibacillus rhamnosus strain 2321
Lacticaseibacillus rhamnosus strain 8966
Lacticaseibacillus rhamnosus strain 2890
Lacticaseibacillus rhamnosus strain 6517
Lacticaseibacillus rhamnosus strain 3034
Lacticaseibacillus rhamnosus strain 2418
Lacticaseibacillus rhamnosus strain 8945
Lacticaseibacillus rhamnosus strain 6519
Lacticaseibacillus rhamnosus strain 6488.
Lacticaseibacillus rhamnosus strain 6470
Lacticaseibacillus rhamnosus strain 6468.
Lacticaseibacillus rhamnosus strain MG4289
Lacticaseibacillus rhamnosus strain 12767
Lacticaseibacillus rhamnosus strain 17246
Lacticaseibacillus rhamnosus strain LDTM7511
Lacticaseibacillus rhamnosus strain 4817
Lacticaseibacillus rhamnosus strain MG4501
Lacticaseibacillus rhamnosus strain MA3
Lacticaseibacillus rhamnosus strain TCP009
Lacticaseibacillus rhamnosus strain 6025
Lacticaseibacillus rhamnosus strain LS8
Lacticaseibacillus rhamnosus strain 2948
Lacticaseibacillus rhamnosus strain HDB1258
Lactobacillus rhamnosus GG strain JL-1
Lacticaseibacillus rhamnosus strain 11132
Lacticaseibacillus rhamnosus strain SMM34
Lacticaseibacillus rhamnosus strain
Lactobacillus rhamnosus strain UNIVASFCAP-Q50
Lacticaseibacillus rhamnosus strain 2770
Lacticaseibacillus rhamnosus strain 2319
Lacticaseibacillus rhamnosus strain 6028
Lactobacillus rhamnosus strain 37503
Lacticaseibacillus rhamnosus strain LNS
Lacticaseibacillus rhamnosus strain 1387
Lacticaseibacillus rhamnosus strain 5960
Lacticaseibacillus rhamnosus strain 5951
Lacticaseibacillus rhamnosus strain 6338
Lacticaseibacillus rhamnosus strain IGM3-9
Lacticaseibacillus rhamnosus strain IGM2-8
Lacticaseibacillus rhamnosus strain Z5
Lacticaseibacillus rhamnosus strain Z23
Lacticaseibacillus rhamnosus strain RHD20
Lactobacillus paracasei strain 221
Lactobacillus paracasei strain 4784
Lactobacillus paracasei strain 4557
Lactobacillus paracasei strain 4432
Lactobacillus paracasei strain 4443
Lactobacillus paracasei strain 4389
Bacterium strain IMAU11894
Lactobacillus paracasei strain 2-56-21
Lactobacillus casei strain NWAFU1550
Lacticaseibacillus paracasei strain 68
Lacticaseibacillus paracasei strain 43
Lacticaseibacillus paracasei strain 36
Lacticaseibacillus paracasei strain 35
Lactobacillus paracasei strain 3319
Lactobacillus casei strain MLG3-30-1
Lactobacillus casei strain MLG19-28
Lactobacillus casei strain MLG19-1
Lactobacillus casei strain SS45.1
Lactobacillus paracasei strain TN-2
Lactobacillus paracasei strain 1876
Lactobacillus paracasei strain 4520
Lactobacillus paracasei strain 4431
Lacticaseibacillus paracasei strain 39
Lacticaseibacillus paracasei strain 38
Lacticaseibacillus paracasei strain 21
Lactobacillus paracasei strain 3133
Lactobacillus paracasei strain 2.90.23
Lacticaseibacillus rhamnosus strainCY12
Lacticaseibacillus rhamnosus strain X253
100
100
97
97
97
97
97
97
97
